# Acute effects of manual breathing assist technique on lung volume and dyspnea in individuals with severe chronic obstructive pulmonary disease: A quasi-experimental study

**DOI:** 10.1097/MD.0000000000039474

**Published:** 2024-08-30

**Authors:** Takako Tanaka, W. Darlene Reid, Mika Laura Nonoyama, Ryo Kozu

**Affiliations:** a Department of Physical Therapy Science, Nagasaki University Graduate School of Biomedical Sciences, Nagasaki, Japan; b Department of Rehabilitation Medicine, Tagami Hospital, Nagasaki, Japan; c Department of Physical Therapy, University of Toronto; KITE – Toronto Rehab-University Health Network; Interdepartmental Division of Critical Care Medicine, University of Toronto, Toronto, Canada; d Faculty of Health Sciences, Ontario Tech University, Ontario, Canada; e Respiratory Therapy & Child Health Evaluative Sciences, The Hospital for Sick Children, Toronto, Canada; f Department of Physical Therapy & Rehabilitation Sciences Institute, University of Toronto, Toronto, Canada.

**Keywords:** chronic obstructive pulmonary disease, dyspnea, lung volume, manual breathing assist technique, oxygenation

## Abstract

**Background:**

Manual breathing assist technique (MBAT) is a common physical therapy technique used to facilitate airway clearance and improve ventilation and oxygenation. The effects during and immediately after intervention in individuals with chronic obstructive pulmonary disease (COPD) are unknown. This study aimed to investigate the acute effects and potential mechanisms of MBAT on lung volume, dyspnea, and oxygenation in individuals with COPD.

**Methods:**

This non-randomized quasi-experimental pre-test/post-test study included participants from pulmonary rehabilitation programs at Tagami Hospital (COPD group) and a community exercise program (Healthy group). During a single session, MBAT was applied during the expiration of every breath for 10 minutes. Dyspnea and lung volumes (tidal volume; *V*_*T*_, inspiratory capacity; IC, inspiratory reserved capacity; IRV, expiratory reserve capacity; ERV) were collected at baseline and after MBAT. Pulse oximetry (SpO_2_), skeletal muscle oxygenation (SmO_2_), and oxy- and deoxy-hemoglobin (O_2_Hb and HHb) using near-infrared spectroscopy (NIRS) were collected at baseline, during, and after MBAT. Between-group comparisons were conducted using the Mann-Whitney U-test and chi-square analyses. Within-group changes before and after MBAT were analyzed using the Wilcoxon signed-rank test. The Kruskal-Wallis test was used to detect differences in NIRS variables in each phase and over time.

**Results:**

Thirty participants with COPD, matched for age and sex, were included, with 15 individuals per group. The difference scores of *V*_*T*_, IRV, and IC were significantly higher in the Healthy group than in the COPD group, but improvements in dyspnea and SpO_2_ were significantly higher in the COPD group. Compared to baseline, ERV decreased significantly in both groups, with dyspnea and SpO_2_ improving significantly only in the COPD group. Inspiratory accessory muscle ΔO_2_Hb and ΔHHb were significantly higher and lower (respectively) during MBAT in the COPD group compared to the Healthy group. Additionally, only the COPD group had increased SmO_2_ during and after MBAT compared to baseline.

**Conclusions:**

MBAT in patients with COPD had acute physiological effects in reducing dyspnea by facilitating expiration and decreasing the recruitment of accessory respiratory muscles. MBAT may help individuals with COPD reduce dyspnea before exercise therapy in a pulmonary rehabilitation program.

## 1. Introduction

Dyspnea is the most problematic symptom for individuals with COPD.^[1]^ It is brought about by the destruction of lung tissue, airways, and alveoli, which compromises gas exchange and causes airflow limitation, particularly on expiration.^[2]^ This ultimately results in a decreased flow of air in and lead to a lower level of oxygen reaching body tissues.^[1]^ Additionally, hyperinflation of the rib cage occurs due to an increase in the functional residual capacity (FRC) and residual volume,^[1]^ which may lead to atrophy of the intercostal muscles and diaphragm. These results further exacerbate dyspnea and impair spontaneous breathing.^[3,4]^ Therefore, interventions aimed at reducing the end-expiratory lung volume (EELV) may alleviate dyspnea in individuals with COPD.

Pulmonary rehabilitation is a non-pharmacological intervention recommended for individuals with COPD.^[1]^ Physical conditioning within a pulmonary rehabilitation program is a crucial component for efficient implementation of the main exercise therapy.^[5]^ One supplemental to conditioning is the manual breathing assist technique (MBAT). MBAT is often referred to as chest wall compression (CWC).^[6]^ Both techniques involve compression of the chest wall synchronously with expiration. CWC is widely used in clinical practice across various respiratory conditions, including COPD, pneumonia, cystic fibrosis, and postoperatively. and has been shown to improve ventilation and airway clearance, particularly when used in conjunction with other physical therapies.^[7]^ In addition, CWC for mechanically ventilated patients has been shown to increase tidal volume (*V*_*T*_) by improving the expiratory flow rate.^[8–10]^ However, MBAT and CWC differ slightly. Both techniques are synchronized with patients’ expiration, with MBAT using lower, more titrated pressures.^[11]^ Compared to CWC this lower and slower pressure has been found to prevent airflow limitation in patients with COPD, resulting in increased inspiratory capacity (IC) and decreased dyspnea.^[12]^ Past studies highlight the effects of MBAT during the procedure. However, there are no reports on MBAT’s impact on lung volume and dyspnea post-treatment. For individuals with COPD, CWC using MBAT may sustain improvement in *V*_*T*_, improve the efficiency of exercise therapy, and provide greater long-term alleviation of dyspnea.

This study aimed to investigate the acute effects and potential mechanisms of MBAT on lung volumes (*V*_*T*_, IC, inspiratory reserved capacity; IRV, expiratory reserve capacity; ERV), dyspnea, and oxygenation in individuals with COPD. We hypothesized that MBAT would improve *V*_*T*_, oxygenation, and dyspnea post-implementation, by reducing ERV.

## 2. Methods

### 2.1. Trial design

This study employed a non-randomized quasi-experimental pre-test/post-test design. It was conducted during a single session at an outpatient clinic within Tagami Hospital in Nagasaki, Japan from December 2017 to March 2020. This study was approved by the Human Ethics Review Committee of Nagasaki University Graduate School of Biomedical Sciences (approval number 17082117) and registered in the clinical trial registry database of the University Hospital Medicine Information Network [UMIN] 000046451).

### 2.2. Participants

Patients diagnosed with COPD (COPD group) were recruited from the pulmonary rehabilitation program at Tagami Hospital in Nagasaki, Japan. Age- and sex-matched healthy older adults (Healthy group) were recruited from a community-dwelling exercise program located in Nagasaki, Japan. Inclusion criteria for the COPD group included a diagnosis of COPD made by a respiratory physician and no acute exacerbation within the preceding 2 months.^[13,14]^ For participants in the Healthy group, participants had no pulmonary disease, which was confirmed with spirometry by the research team. Participants in both groups were excluded if they were unable to lie supine due to kyphosis or a history of spinal compression or if they had rib fractures. Furthermore, those who did not consent to participate in the study were excluded.

### 2.3. Study protocol

#### 2.3.1. Procedure

The procedure started with a baseline period of at least 10 minutes of rest and quiet breathing. During this time, pulse rate (PR), oxygen saturation (SpO_2_), respiratory muscle oxygenation, and respiratory rate were monitored to ensure a steady state. After the baseline period, MBAT was performed for 10 minutes, followed by another 5-minute period of rest and quiet breathing. The participants were instructed to refrain from performing any special breathing techniques, such as pursed lips, during this interval.

#### 2.3.2. MBAT protocol

**Figure 1.**
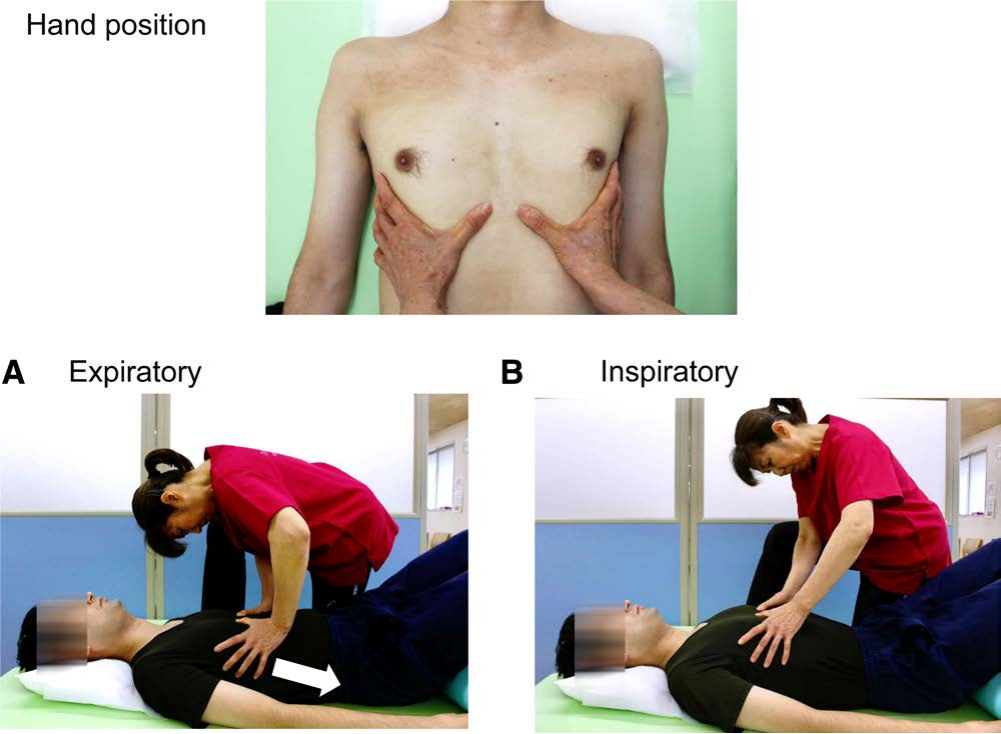
Application of manual breathing assist technique (MBAT). During MBAT, the hands of both physiotherapists were positioned on the subject’s lower rib cage (upper part of the costal arch). (A) Expiration: The physiotherapist applies slow and gentle compression to the rib cage synchronously with expiration. The direction of compression should be in the caudal and dorsal directions, keeping in mind the physiological direction of lower thoracic movement (yellow arrow). (B) Inspiration; a physiotherapist maintains hand-to-chest wall contact during inspiration; no pressure was applied.

The physiotherapist was positioned on their side, with the participants in the supine position. All MBAT maneuvers were performed with the participants in the supine position. Previous studies have identified the supine position as optimal due to its ability to produce the lowest functional residual capacity,^[15]^ and facilitated expiration during the MBAT maneuver.^[16]^ She then placed her hands bilaterally on the anterior lower rib cage (the upper part of the costal arch). The physiotherapist applied slow and gentle compression to the rib cage synchronously with each participant’s expiration from beginning to end. Compression was applied in the caudal and dorsal directions and synchrony with the lower thoracic movement. The strength of the compressions was adjusted to the participants in which they were most comfortable based on the interview feedback. Hand-to-chest wall contact was maintained during inspiration; however, compression was released immediately as the participant began to inspire. The speed and frequency of MBAT varied according to the participant’s respiratory rate during the 10-minute procedure. To minimize inter-therapist variability, the MBAT was performed by the same physiotherapist (T. T.) with 25 years of experience using this technique (Fig. 1; see Video, Supplemental Digital Content, which demonstrates the manual breathing assist technique; MBAT, http://links.lww.com/MD/N460). Informed consent was obtained from the collaborating staff regarding the publication of this photograph and video.

### 2.4. Outcome measures

The Demographics of all the study participants were obtained prior to the MBAT protocol. This included age and sex. In addition, baseline spirometry (percent predicted forced vital capacity (%FVC), percent predicted forced expiratory volume in the first second (%FEV_1_), and the ratio of the forced expiratory volume in the first 1s to the forced vital capacity (FEV_1_/FVC), dyspnea, SpO_2,_ and PR were collected.

#### 2.4.1. Lung volumes

Lung volumes (*V*_*T*_, ERV, IRV, and IC) were measured before and immediately after the 10-minute MBAT maneuver using a portable automatic calibrated spirometer (Autospiro AS-507^®^; MINATO MEDICAL SCIENCE, Osaka, Japan). Each participant repeated the maneuvers until 3 reliable tracings were obtained, and the highest values were retained for analysis.^[17,18]^ Measurements were expressed in absolute values and as a percentage of the predicted value in a healthy Japanese population.^[19]^

#### 2.4.2. Dyspnea

Dyspnea sensation was evaluated using a modified Borg 10-point rating scale^[20]^ before and immediately after MBAT.

#### 2.4.3. Oxygenation and pulse rate

SpO_2_, PR, and accessory inspiratory muscle oxygenation were measured continuously throughout the study protocol. A pulse oximeter (PULSOX-ME300^®^; TEIJIN, Tokyo, Japan) was used to measure SpO_2_ and PR. An accessory inspiratory muscle oxygenation was measured with spatially resolved near-infrared spectroscopy (NIRS) (Oxysoft® Artinis Medical Systems, BV, Netherlands), using the sternocleidomastoid muscle (SCM) as the key accessory muscle.^[21,22]^ The SCM was selected because it is actively recruited to assist the primary inspiratory muscles when ventilatory loads are increased,^[23]^ thus indicating ventilatory effort/work of breathing. Three indicators of accessory inspiratory muscle oxygenation were collected. Skeletal muscle oxygen saturation (SmO_2_) is an important measure of local tissue oxygen delivery and utilization because it provides information on the muscle’s capacity to match oxygen supply relative to metabolic demand.^[24–26]^ Oxyhemoglobin (O_2_Hb) and deoxyhemoglobin (HHb) are an index of respiratory muscle oxygen extraction.^[27]^ The NIRS device was placed over the middle third of the right SCM belly between the mastoid process and sternal notch. The right tibialis anterior (TA) muscle served as the control muscle. TA was selected as the control muscle because it is the furthest from the respiratory muscles and is not associated with respiratory or accessory muscles to prove that the changes in SCM O_2_Hb and HHb were due to a decrease in ventilatory effort, according to previous studies.^[23]^ NIRS variables were zeroed at the beginning of the MBAT protocol and data were acquired at 10 Hz. Throughout the protocol, changes in SCM and TA O_2_Hb and HHb were directly measured, and the software was used to calculate change (Δ) of total hemoglobin (Δ*t*Hb = ΔO_2_Hb + ΔHHb).

### 2.5. Sample size

We were unable to find evidence of an acute effect of MBAT on *V*_*T*_, dyspnea, or accessory inspiratory muscle oxygenation in patients with COPD. We estimated the sample size from a study assessing the effect of chest wall stretching on *V*_*T*_ by Putt et al.^[28]^ For an effect size of 1.2, an alpha of <0.05, and a power of 80%, it was estimated that 15 participants per group were required.

### 2.6. Statistical analysis

All data are expressed as median (interquartile range) unless otherwise described. Between groups (COPD vs Healthy group) were compared using the Mann–Whitney *U* test and chi-square analyses. The Wilcoxon signed-rank test was used to analyze before and after MBAT changes within the groups (lung volumes, dyspnea, SpO_2_, and PR). To analyze NIRS variables over time and within groups, the mean change for each phase was used. The Kruskal–Wallis test was used to detect differences in ΔO_2_Hb, ΔHHb, and Δ*t*Hb in each phase and over time (from baseline to MBAT to recovery). Dunnett’s method was used as a post hoc test. The level of statistical significance for all analyses was set at a probability of <5%. Statistical analysis was performed using IBM SPSS statistics version 25.0 for Windows (IBM Corporation, Armonk).

## 3. Results

**Figure 2.**
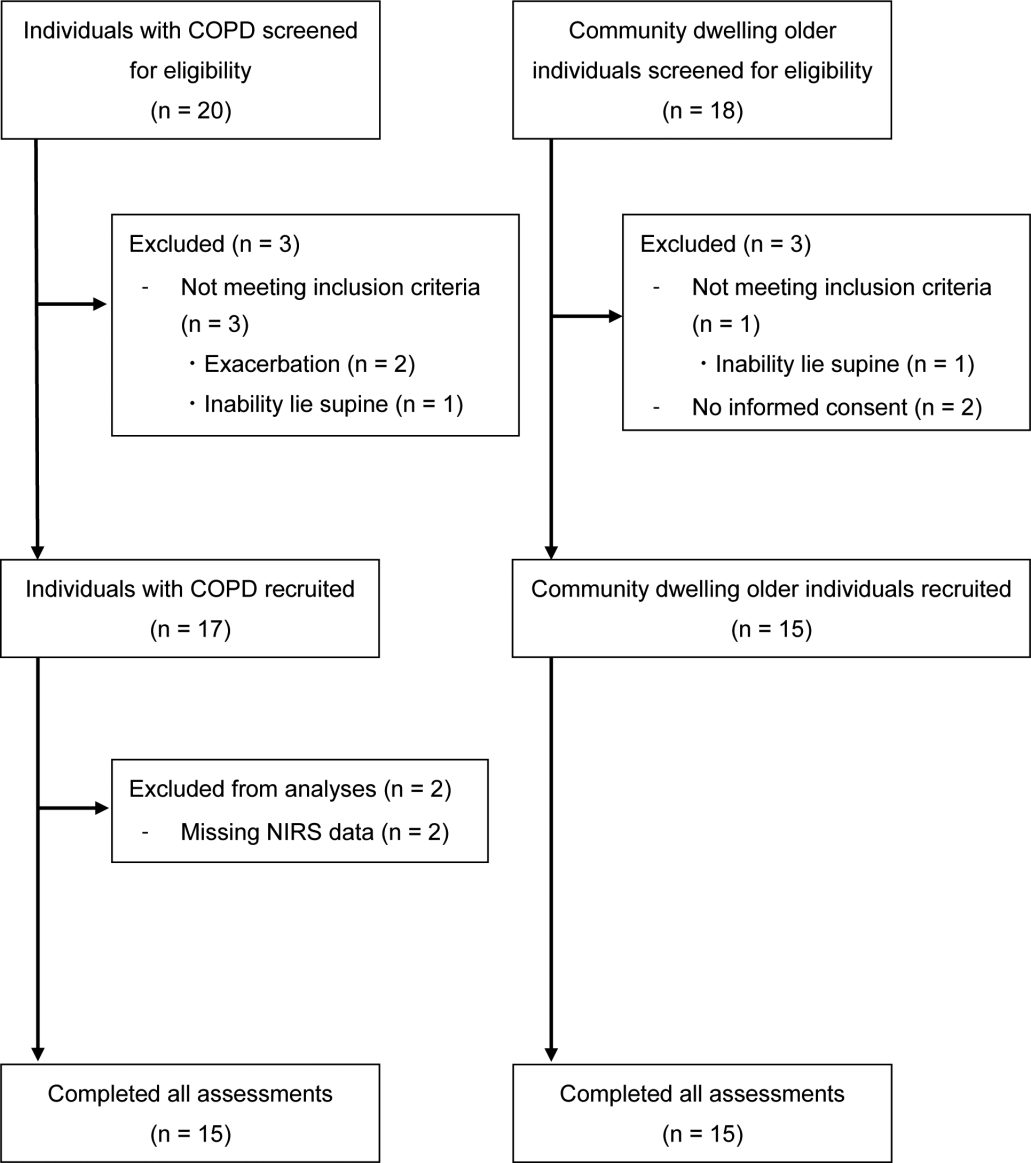
Flow diagram of study participants. COPD = chronic obstructive pulmonary disease.

A total of 20 individuals with COPD and 18 community-dwelling older adults were screened for eligibility based on predetermined criteria. Of these, 17 participants with COPD and 15 healthy community-dwelling older adults were recruited. Data from 2 participants with COPD were excluded from the analyses because of missing data, as shown in Figure 2. No adverse events or complications were observed during the study period.

### 3.1. Participant characteristics

The participant characteristics are described in Table 1. The COPD group exhibited significantly lower BMI, %FVC, %FEV_1_, and FEV_1_/FVC (*P* < .05, .01, .001, and .001, respectively) than the Healthy group. Additionally, the COPD group demonstrated higher dyspnea scores (modified Borg 10-point rating scale) and PR at baseline, during rest (*P* < .01, respectively) compared to the Healthy group.

**Table 1:** Participant characteristics.

	COPD group (n = 15)	Healthy group (n = 15)	*P* value
Age (yr)	77 (69–92)	79 (70–91)	.635
Male (% males)	13 (87%)	7 (70%)	.582
BMI (kg/m^2^)	21.4 (18.2–23.0)	24.8 (23.5–26.9)	.05
%FVC (% predicted)	60 (57–98)	88 (80–106)	.01
FEV_1_/FVC (%)	45 (19–68)	84 (80–90)	.001
%FEV_1_ (% predicted)	42 (31–49)	88 (80–96)	.001
Borg 10-point rating scale at rest	3 (2–4)	0 (0–0)	.01
SpO_2_ (%)	96 (95–97)	97 (96–97)	.816
Pulse rate (bpm)	84 (65–96)	68 (51–75)	.01

Data are presented as median (interquartile range) unless stated.

%FEV_1_ = forced expiratory volume on the first second expressed as a percentage of predicted, %FVC = forced vital capacity expressed as a percentage of predicted, BMI = body mass index, COPD = chronic obstructive pulmonary disease, SpO_2_ = pulse oxygen saturation.

### 3.2. Lung volumes, dyspnea, and oxygenation

The pre-/post-MBAT between-group differences in *V*_*T*_, IRV, and IC were significantly higher in the healthy group than in the COPD group (Table 2). Conversely, the differences in the Borg 10-point rating scale, SpO_2_, and PR were significantly higher in the COPD group compared to the healthy group. Following MBAT, both groups exhibited significantly increased *V*_*T*_ and IC, and a decrease in ERV compared to baseline (*P* < .01; Table 2). Additionally, IRV significantly increased in the healthy group (*P* < .01), whereas it remained unchanged in the COPD group. The PR and Borg 10-point rating scale significantly decreased (*P* < .05,.01), and SpO_2_ significantly increased in the COPD group after MBAT compared to baseline (*P* < .05). These variables did not change over time in the healthy group.

**Table 2:** Changes in lung volumes, dyspnea, and oxygenation by MBAT.

	COPD group			Healthy group			Between-group differences
	Before	After	Differences	Before	After	Differences	
*V*_*T*_ (L)	0.52 (0.46–0.54)	0.86 (0.78–0.92)	0.34** (0.32–0.38)	0.60 (0.57–0.63)	1.24 (1.09–1.30)	0.64** (0.42–0.67)	0.30*** (0.29–0.64)
ERV (L)	0.64 (0.56–0.83)	0.30 (0.31–0.40)	0.34**(0.21–0.41)	0.75 (0.62–0.80)	0.37 (0.33–0.42)	0.38** (0.38–0.42)	0.03 (0.02–0.04)
IRV (L)	0.82 (0.58–0.90)	0.99 (0.79–1.03)	0.17 (0.13–0.21)	1.06 (0.93–1.26)	1.51 (1.00–1.69)	0.45** (0.08–0.48)	0.37**** (0.06–0.40)
IC (L)	1.51 (1.08–1.59)	1.77 (1.20–1.93)	0.26** (0.01–0.34)	2.01 (1.62–2.39)	2.43 (1.89–2.61)	0.42** (0.22–0.48)	0.16*** (0.11–0.22)
Borg 10-point rating scale	3 (2–4)	2 (1–2)	1**(1–2)	0 (0–0)	0 (0–0)	0 (0–0)	1*** (1–2)
SpO_2_ (%)	96 (95–97)	98 (97–98)	2* (1–2)	97 (96–97)	97 (97–97)	0 (0–1)	2*** (1–2)
Pulse rate (bpm)	84 (65–96)	74 (71–81)	10* (6–15)	68 (57–73)	66 (58–70)	2 (1–3)	7**** (5–12)

Data are presented as median (interquartile range) unless stated.

COPD = chronic obstructive pulmonary disease, ERV = expiratory reserve volume, IC = inspiratory capacity, IRV = inspiratory reserve volume, MBAT = manual breathing assist techniques, SpO_2_ = pulse oxygen saturation, *V*_*T*_ = tidal volume.

* Significant change between before and after MBAT within the group (*P* < .05).

** Significant change between before and after MBAT within the group (*P*< < .01)

*** Significant change between COPD and healthy group (*P* < .05).

**** Significant change between COPD and healthy group (*P*< < .01).

**Figure 3.**
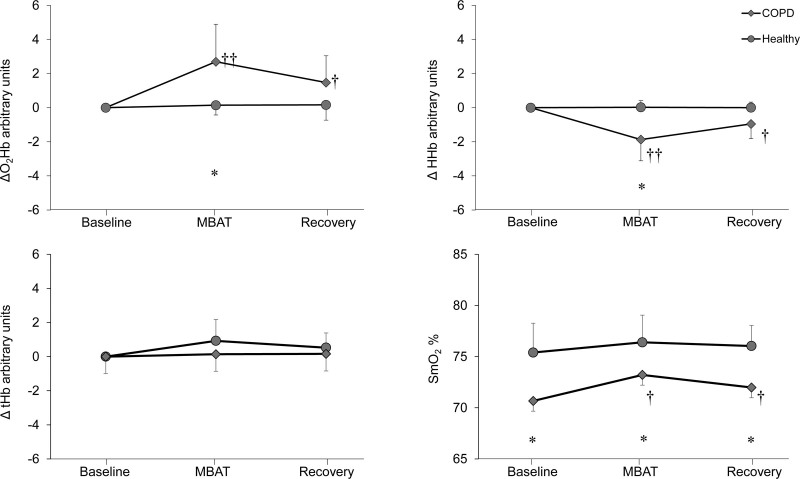
Sternocleidomastoid muscle (SCM) oxygenation responses. Changes in oxygenated (ΔO_2_Hb), deoxygenated hemoglobin (ΔHHb), total hemoglobin (Δ*t*Hb), and skeletal muscle oxygen saturation (SmO_2_) of the sternocleidomastoid (SCM) during and after the manual breathing assist technique (MBAT). Diamonds = COPD group; circles = Health group. Means ± SD are shown. * *P* < .05, significant differences between the COPD and Healthy groups. †*P* < .05, ††*P* < .01 indicates significant differences compared to the baseline. COPD = chronic obstructive pulmonary disease, MBAT = manual breathing assist technique, SCM = sternocleidomastoid muscle.

Figure 3 shows the SCM oxygenation response. In the between-group comparisons, ΔO_2_Hb, and ΔHHb were significantly higher and lower (respectively) during MBAT in the COPD group compared to the healthy group. Δ*t*Hb showed no differences between both groups across any phase. SmO_2_ was significantly lower in the COPD group than in the healthy group in all phases (Baseline; 70.7 ± 3.1% vs 75.4 ± 2.9%, MBAT; 73.2 ± 2.9% vs 76.4 ± 2.7%, Recovery; 72.0 ± 3.1% vs 76.0 ± 2.0%, *P* < .05, respectively). For within-group comparisons, the COPD group exhibited significantly increased ΔO_2_Hb (2.70 ± 2.20 µm) and significantly decreased ΔHHb (1.87 ± 0.02 µm) during MBAT compared to baseline (*P* < .01). SmO_2_ significantly increased from 70.7 ± 3.4 to 73.2 ± 2.9% (*P* < .01). These differences persisted during recovery, although the magnitude was less (ΔO_2_Hb; 1.47 ± 1.57 µm, ΔHHb 0.95 ± 0.87 µm, SmO_2_ 72.0 ± 3.1%, *P* < .05, respectively). The Healthy group showed no changes in NIRS outcomes during baseline, MBAT, and recovery. In addition, ΔtHb remained unchanged in both groups.

## 4. Discussion

To our knowledge, this is the first study to investigate the acute effects of MBAT on lung volume and dyspnea in individuals with COPD compared with healthy controls. Although the ERV decreased by similar amounts in both groups, there was a more pronounced effect in the COPD group, as evidenced by a more significant reduction in dyspnea, reduced respiratory accessory muscle recruitment, and improvement in oxygenation observed exclusively in this group. These results support our hypothesis that MBAT would improve *V*_*T*_, oxygenation, and dyspnea post-implementation, by reducing ERV.

The current study showed that ERV decreased, and VT and IC significantly increased in both groups after MBAT. The effect of MBAT on lung volume might be attributed to improved chest wall compliance and/or increased passive expiratory flow, even post-treatment. While no previous studies specifically addressed the acute effects of MBAT on lung volume, there are published reports on CWC that support our findings. Several studies have demonstrated that compressive forces on the lower rib cage during CWC increase the intrapleural or abdominal pressure, leading to an enhanced expiratory flow rate. This was shown in various populations, including healthy subjects,^[16]^ mechanically ventilated patients,^[6,8,9]^ individuals with COPD^[29,30]^ and cystic pulmonary fibrosis.^[31]^ Furthermore, in individuals with COPD, hyperinflation of the rib cage, changes in diaphragmatic function, and increased contribution of accessory inspiratory muscles toward chest wall motion have been associated with airflow obstruction.^[32–34]^ Finally, another study indicated that the chest wall muscle stretching technique was associated with increased vital capacity in COPD patients.^[28]^

Moreover, SpO_2_ significantly increased after MBAT in the COPD group compared to that in the healthy group. This contrasts with some previous studies, which reported oxygenation increased during CWC but returned to pretreatment levels as soon as chest compressions were discontinued.^[35]^ Similarly, another study showed improvements in muscle oxygenation during a hyperventilation exercise in normocapnic COPD patients.^[36]^ The differences between our results and those of previous studies may be attributed to variations in the participants (e.g., non-mechanically ventilated patients) or the procedures being performed (e.g., MBAT vs CWC).

Concerning accessory inspiratory muscle oxygenation, the COPD group exhibited significant increases in ΔO_2_Hb, ΔHHb, and SmO_2_ during MBAT compared with baseline, and these differences persisted during recovery. Conversely, the healthy group showed no changes in NIRS outcomes during baseline, MBAT, and recovery. Between-group comparisons showed ΔO_2_Hb and ΔHHb were significantly higher and lower (respectively) during the last 2 phases of MBAT in the COPD group compared to the Healthy group. SmO_2_ levels were significantly lower in the COPD group at all phases. Additionally, we found no changes in the oxygenation of the TA control muscle in either group. These improvements in respiratory accessory muscle oxygen extraction show a better balance between local tissue oxygen delivery and utilization in the COPD group. Decreased SCM recruitment due to MBAT may reduce the oxygen demand while maintaining blood flow (ΔtHb) in patients with COPD. No previous studies have investigated inspiratory accessory muscle oxygenation for therapeutic efficacy in patients with COPD at rest. However, earlier studies using NIRS during incremental inspiratory threshold loading reported progressively increasing SCM ΔHHb preceding task failure in healthy adults^[23,37]^ and moderate to severe COPD patients.^[22]^ Katayama et al^[38]^ reported that SCM HHb levels were increased in healthy men with voluntary hyperpnea. This suggests that there was no muscle contraction in the TA or SCM of healthy subjects because the work of breathing, which is expressed as ventilatory effort, was not higher in healthy subjects than in COPD patients.

In our study, MBAT had an acute effect on lung volume. However, the physiological mechanisms differ between the COPD and healthy groups. In COPD individuals, MBAT chest compression maneuvers significantly affected the inward recovery of the chest wall by promoting expiration, thereby decreasing ERV. Therefore, it can be inferred that dyspnea was alleviated by an increase in *V*_*T*_ with reduced SCM recruitment during the next inspiration. In healthy individuals, MBAT also led to a decrease in ERV. However, unlike in the COPD group, it can be assumed that both the ERV decrease and IRV increase contributed to an increase in *V*_*T*_. Dyspnea did not significantly change for this group, likely because of less accessory respiratory muscle recruitment compared to the COPD group.

The strengths of our study demonstrated MBAT as a potential conditioning maneuver to reduce dyspnea in individuals with COPD participating in a pulmonary rehabilitation program and included healthy controls and utilized validated and objective measures of lung volume, oxygenation, and dyspnea. However, this study has some limitations. Firstly, MBAT was conducted during quiet breathing in a supine position, and its effects may differ in other positions. Secondly, lung volumes such as ERV, IRV, and IC were not measured using the gold-standard methods of whole-body plethysmography.^[39]^ However, considering the timing of the MBAT protocol and the practical considerations regarding equipment size and cost, we believe that portable spirometry is more suitable in clinical settings. Thirdly, although NIRS has been utilized in previous studies to assess accessory muscle oxygenation and deoxygenation,^[22,27,38,40]^ we assessed SCM activity using NIRS and did not employ electromyography to directly measure muscle activation. Fourthly, although MBAT was administrated by an experienced physiotherapist, results may be varied among therapists. Evaluating these mechanisms in a larger sample of COPD patients with more than 1 physiotherapist would enhance both internal and external validity. Fifthly, although our study compared outcomes between COPD and healthy groups, it did not include a control intervention. A randomized controlled trial comparing interventions and incorporating the blinding of participants, assessors, and data analysts is recommended for future research. Lastly, although some improvements in outcomes were statistically significant in the COPD group, the clinical relevance of these effects may be limited because of their small magnitude. Given the noninvasive, simple, and quick nature of this intervention, future research should investigate the long-term effectiveness, acceptance, and importance of MBAT in individuals with COPD.

## 5. Conclusion

MBAT resulted in improvements in dyspnea and oxygenation (SpO_2_ and SmO_2_) in individuals with COPD, both during and immediately post-treatment. The mechanism of this effect is likely an increase in *V*_*T*_ and IC, resulting in a reduction in ERV. MBAT is a commonly used, noninvasive, simple, and quick intervention that can be implemented. Its use before exercise in individuals with COPD holds promise in alleviating dyspnea and enhancing the efficacy of exercise therapy.

## Acknowledgments

We thank all participants for their participation. We are also very appreciative of the valuable assistance provided by our colleagues, who are physiotherapists at Tagami Hospital, during data collection in this study. We also thank Dr Sumihisa Honda (Nagasaki University Graduate School of Biomedical Sciences) for his statistical advice.

## Author contributions

**Conceptualization:** Takako Tanaka, Ryo Kozu.

**Data curation:** Takako Tanaka, Ryo Kozu.

**Formal analysis:** Takako Tanaka, W. Darlene Reid.

**Funding acquisition:** Takako Tanaka.

**Investigation:** Takako Tanaka.

**Methodology:** Takako Tanaka, Ryo Kozu.

**Project administration:** Takako Tanaka, W. Darlene Reid, Mika Laura Nonoyama, Ryo Kozu.

**Resources:** Takako Tanaka.

**Software:** Takako Tanaka.

**Supervision:** Takako Tanaka, W. Darlene Reid, Mika Laura Nonoyama, Ryo Kozu.

**Validation:** Takako Tanaka, W. Darlene Reid, Mika Laura Nonoyama, Ryo Kozu.

**Visualization:** Takako Tanaka, W. Darlene Reid, Mika Laura Nonoyama, Ryo Kozu.

**Writing – original draft:** Takako Tanaka, W. Darlene Reid, Mika Laura Nonoyama, Ryo Kozu.

**Writing – review & editing:** Takako Tanaka, W. Darlene Reid, Mika Laura Nonoyama, Ryo Kozu.

## Supplementary Material


